# Measuring value in health care: lessons from accountable care organizations

**DOI:** 10.1093/haschl/qxae028

**Published:** 2024-03-01

**Authors:** Chenzhang Bao, Indranil R Bardhan

**Affiliations:** Department of Management Science and Information Systems, Oklahoma State University, Tulsa, OK 74106, United States; Department of Information, Risk, and Operations Management, Red McCombs School of Business, The University of Texas at Austin, Austin, TX 78712, United States

**Keywords:** accountable care organizations, health care value, social determinants of health, data envelopment analysis, care coordination

## Abstract

Accountable care organizations (ACOs) were created to promote health care value by improving health outcomes while curbing health care expenditures. Although a decade has passed, the value of care delivered by ACOs is yet to be fully understood. We proposed a novel measure of health care value using data envelopment analysis and examined its association with ACO organizational characteristics and social determinants of health (SDOH). We observed that the value of care delivered by ACOs stagnated in recent years, which may be partially attributed to challenges in care continuity and coordination across providers. ACOs that were solely led by physicians and included more participating entities exhibited lower value, highlighting the role of coordination across ACO networks. Furthermore, SDOH factors, such as economic well-being, healthy food consumption, and access to health resources, were significant predictors of ACO value. Our findings suggest a “skinny in scale, broad in scope” approach for ACOs to improve the value of care. Health care policy should also incentivize ACOs to work with local communities and enhance care coordination of vulnerable patient populations across siloed and disparate care delivery systems.

## Introduction

For over a decade, the US health care system has witnessed a shift toward value-based care models, among which the accountable care organization (ACO) program is the flagship initiative. Accountable care organizations represent groups of doctors, hospitals, and other health care providers that join voluntarily to provide coordinated, patient-centric care. They are rewarded with a portion of cost savings if they lower health expenditures below a predefined benchmark, while maintaining or improving the quality of health care services. A decade has passed since the inception of the Medicare Shared Savings Program (MSSP) in 2012, one of the largest ACO programs, yet little is known about the value of care delivered by ACOs.

Prior studies have examined ACO performance along various dimensions, such as health spending, patient satisfaction, and readmissions, but reported mixed results.^1-5^ However, in health care, value is not simply about cost reduction or quality improvement. Instead, it is defined as “health outcomes achieved per dollar spent,” which must encompass the multi-input, multi-output nature of health care delivery.^6-8^ This notion indicates that health care value should be a composite measure that combines multiple, often conflicting, performance dimensions, instead of individual measures. Indeed, researchers have recognized that “if the real goal of value-based health care were cost reduction, pain killers and compassion would be sufficient….”^9^ Similarly, quality-improvement efforts may not necessarily translate to greater value because they may prioritize operational overhauls.

Furthermore, recent research suggests that the incentive structure of ACOs may not resolve cost–quality tradeoffs, and shared savings (rewards) are not always provided to the most cost-efficient performers.^10,11^ This is particularly worrisome since ACOs that earned rewards were less likely to exit the MSSP, which may result in lemon ACOs staying in the program.^12^ Thus, the extant evidence on health care value has been inconclusive in ACOs. Specifically, it remains unclear whether ACOs have been successful in delivering high-value health care, and what the value drivers and opportunities for improvement are. Considering the significant investments made by the Centers for Medicare and Medicaid Services (CMS) in value-based care initiatives, it is critically important to develop a better understanding of health care value in ACOs.

Our research objectives are 2-fold. First, we propose and implement a novel approach to quantify health care value using data envelopment analysis (DEA), an optimization-based approach that measures the relative effectiveness of ACOs in utilizing input resources to improve patient outcomes. We study trends in ACO value using publicly available data from a national sample of MSSP ACOs and highlight improvement opportunities for underperforming ACOs. Specifically, we identify shortfalls in health outcomes of their assigned patient population or resource utilization, compared with their peers. Second, we conduct econometric analyses to identify explanatory factors associated with ACO health care value, specifically focusing on the role of ACO organizational characteristics and social determinants of health (SDOH) factors.

## Data and methods

### Data sources and study population

We combined multiple databases to create a national sample of 865 distinct ACOs enrolled in the MSSP between 2013 and 2021. First, we collected the MSSP public use files maintained by CMS. This is a commonly used data source in the literature to obtain longitudinal data on ACO-specific characteristics and operational performance, such as the number of assigned beneficiaries, contract start date, enrolled risk model, health expenditures, and quality metrics.^12,13^ This database also reports each ACO's participant list based on their Taxpayer Identification Number or CMS Certification Number and service counties of ACO beneficiaries.^14^

Next, we matched participating entities with publicly reported data from each ACO's website to identify their leadership type—that is, whether the ACO was managed by a hospital or physician group. We cross-validated our classification with ACO taxonomy from the Leavitt Partners ACO Database to ensure accuracy. Last, we collected SDOH data for the ACOs’ service counties and aggregated them to the ACO level, weighted by the number of assigned beneficiaries in each county. Specifically, SDOH factors on economic well-being and transportation convenience were obtained from the American Community Survey, food-access data from the Food Environment Atlas, and health resource availability from the Dartmouth Atlas of Healthcare and the Healthcare Information and Management Systems Society.

The unit of analysis in our study was an ACO-year observation. We eliminated 10 observations with missing values in the key variables to conduct meaningful analyses. Our final dataset consists of 3850 ACO-year observations.

### Measuring health care value

Researchers have resorted to several approaches to infer health care value, including stochastic frontier estimation, quadrant diagrams, weighted composite scores, and regression analysis.^15^ However, these methods are subject to significant methodological limitations, such as the need for standard weights and a priori knowledge about production function, which may explain their limited use in practice. (In online Appendix section A, we have discussed these methodological limitations and the empirical advantages of DEA in detail.) In this study, we propose DEA as an alternate approach to measure health care value, which offers benefits to overcome these limitations.^16^

Data envelopment analysis is a nonparametric optimization approach that uses linear programming techniques to determine the optimal efficiency of decision-making entities, where each entity converts a predetermined set of inputs into outputs.^6^ We use DEA to study the production of patient health outcomes (ie, outputs) using clinical resources as inputs.^8^ The DEA model identifies a Pareto frontier of “best-performing ACOs” that utilize the least amount of input resources to yield the highest outcomes. Data envelopment analysis allows each ACO to benchmark itself against its “highest value” peers with inputs and outputs that are at least equal to or better than the focal ACO. By comparing the focal ACO with its peers, we then calculate its value score based on the extent to which the ACO optimizes its resource allocation to achieve the best possible outcomes. The ACO value score is a continuous number ranging from 0 to 1, with higher scores indicating greater health care value relative to peer ACOs.

We utilized the slacks-based measure (SBM) DEA model to quantify ACO value.^17^ The SBM model takes into consideration excess input resources and shortfalls in outputs, also termed “slacks”. It minimizes input resources while simultaneously maximizing quality outcomes, which is aligned with ACO objectives. The input set includes the major types of clinical resources, such as operating expenses, capital expenses, and staffing levels for primary care physicians, specialists, and other clinicians.^18,19^ We normalized input variables to a beneficiary-year basis to account for ACO scale effects.^20^

We considered 4 domains of quality outcomes in the DEA outputs: patient/caregiver experience, care coordination/patient safety, preventive health, and at-risk population. The CMS evaluates ACO quality using a comprehensive set of over 30 individual measures that span these domains. We selected those measures that were part of the pay-for-performance (P4P) evaluation method and used the same approach as CMS to aggregate them into 4 composite scores of quality outputs.^12^ A detailed discussion of our DEA model is available in section A of the online Appendix, along with an illustrative example in section B to describe the intuition behind the DEA model. We present additional details of DEA inputs and outputs in section C.

### Regression analysis

We used a multivariate linear regression model to identify the factors associated with ACO health care value. Specifically, we regressed the ACO value score (calculated using DEA) on ACO characteristics that may affect their operations and performance. The ACO characteristics included leadership taxonomy (hospital-managed vs physician-led), the number of years of MSSP enrollment, participation in 2-sided risk tracks, upfront investment, the numbers of ACO beneficiaries and distinct participants, weighted Hierarchical Condition Category risk score by beneficiary type (the weighted HCC risk score should not be confused with at-risk population; the former is a covariate in regression analysis while the latter is a quality domain and thus treated as a DEA output), a Herfindahl-Hirschman Index of beneficiary concentration in service counties, and the proportion of health expenditures on home health agency.^14,20,21^ In our sample of 865 distinct ACOs, 135 changed leadership taxonomy and 158 switched their risk track. In other words, our regression analysis accounts for these time-variant ACO characteristics.

We also sought to understand regional differences in ACO value scores.^13,20^ Hence, we used various SDOH factors based on ACO service areas and examined their association with ACO value. Specifically, we used unemployment rate and median household income as proxies to measure regional economic well-being. For transportation convenience, we considered public transportation for commuting among the workforce and the average commute time as suitable proxies. With regard to healthy food access, we used the ratio of low-income population with grocery store access and per capita expenditures on fast food. Last, we included 3 measures of community health resource availability: hospital-based registered nurse to bed ratio, fulfillment of composite annual primary care checkups, and the average distance between local health care organizations. These SDOH factors were collected at the county level and aggregated to the ACO-year unit using the number of beneficiaries in each county as weights. We provide a detailed description of each variable in section D of the online Appendix and regression model specification in section E.

Our regression model included ACO fixed effects to account for time-invariant, ACO-specific effects. We also added year fixed effects to address changes in MSSP regulations and macroeconomic shocks applicable to all ACOs. Since CMS started publishing data on ACO service counties in 2014, we were unable to construct SDOH factors for 2013. Hence, our regression analysis used a sample of 3628 ACO-year observations from 2014 to 2021.

### Limitations

Our study has several limitations. First, our results should not be interpreted as causal because some strategic decisions, such as leadership type and risk track, were not made in a random manner and may be subject to performance considerations.^4^ Second, our analyses focused on MSSP ACOs and did not represent ACOs that served non-Medicare patient populations. However, our proposed framework to quantify health care value using DEA can be generalized to other settings. Third, we used P4P quality measures to construct DEA outputs because CMS did not publish quality benchmarks for pay-for-reporting measures. Future research can include these measures to build a more holistic assessment of health care value. Fourth, the MSSP experienced several changes in recent years, such as categorizing low-/high-revenue ACOs and transitioning to electronic clinical quality measure (eCQM) reporting. We were not able to test their effects on health care value due to a significant loss of observations in our analyses.

Furthermore, we were unable to adjust the number of distinct participants based on organization type. While our results are consistent when we controlled for the number of individual physicians and hospitals, we acknowledge this data limitation and call for future studies to obtain a more accurate estimate of the impact of ACO size. Last, our SDOH data were collected at the county level where ACO beneficiaries resided.^22^ This arguably introduced noise into our model, although such noise was mostly random and should not qualitatively alter our findings since patient service locations were not directly under ACO control. Further analyses using patient-level data can significantly improve the overall analyses.

## Results

### Health care value

**Figure 1.**
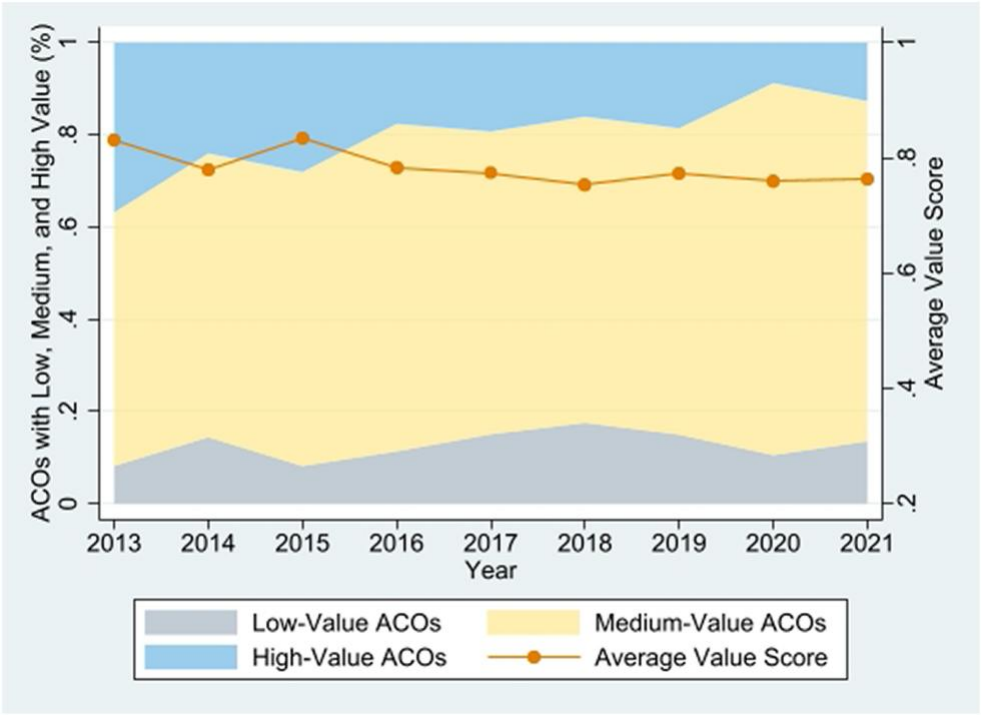
Trends in health care value of accountable care organizations (ACOs), 2013–2021. Source: Authors’ analysis of data from the Medicare Shared Savings Program public use files from 2013 to 2021. One standard deviation above and below average were used as thresholds to determine high and low value, respectively.

We first describe ACO health care value based on the DEA model. Only 18% of ACOs achieved a value score of 1. These ACOs were the best-value performers on the Pareto optimal frontier and rated as 100% effective in their utilization of input resources to generate outcomes, relative to other ACOs. The average value scores fluctuated around 0.8 during the early years and stagnated at the level of 0.76, indicating that, on average, ACOs have the potential to improve their value of care delivery by 24%, by reducing expenses and increasing P4P quality performance measures (Figure 1).

We classified ACOs into 3 groups—low, medium, and high value—based on their mean value scores and standard deviation. The colored area in Figure 1 represents the size of each group. In 2013, 81 (37%) ACOs had a high-value score, compared to only 59 (12.7%) ACOs in 2021. In contrast, the proportion of medium-value ACOs increased from 55% to 73%, indicating a growing number of ACOs that delivered health care at medium value.

### Gaps between current and optimal performance

**Figure 2.**
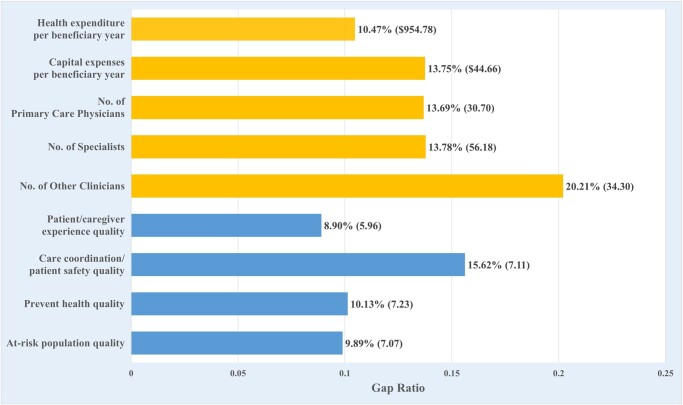
Gaps between current and optimal levels of clinical resources and quality outcomes in accountable care organizations (ACOs), 2013–2021. Source: Authors’ analysis of data from the Medicare Shared Savings Program public use files from 2013 to 2021. Gap is defined as the difference between current and optimal levels in ACO clinical resources (inputs) and quality outcomes (outputs). Gap ratio is the normalized gap value over the current performance level. The length of the bar represents the gap ratio with the gap value in the parentheses. The yellow bar represents ACO inputs while the blue bar represents ACO quality outcomes.

Next, we examined the DEA slacks, which measure the gap between current and optimal levels of inputs and outputs (Figure 2). This allowed us to understand the extent of reduction in resource consumption and improvement in quality outcomes that an ACO must undertake to deliver high-value health care. On the input side, the stagnation in health care value can be attributed to inefficient use of clinical resources, with an average slack of 10.5% between the current operating expenses and optimal level, and an average slack of 13.8% for capital expenses. Furthermore, we observed an average slack of 13.7% in the number of primary care physicians and specialists, and a 20.2% slack for other clinicians. For an average ACO, this amounts to a total of $1000 in cost savings per beneficiary-year and a reduction of 120 clinicians, without compromising quality outcomes.

On the output side, the care coordination/patient safety domain exhibited the most prominent slack. On average, ACOs can increase quality scores in this domain by 7.1 percentage points, which corresponds to a 15.6% improvement from the mean. Since ACOs already performed well in other quality domains, the extent of possible improvements was relatively modest, ranging from an improvement opportunity of 10.1% for preventive health to 8.9% for patient/caregiver experience.

### Peer ACO comparison

At the individual ACO level, DEA can help managers identify peer ACOs whose relative performance can be used as the ideal benchmark. In other words, benchmarking based on comparison against peer ACOs on the Pareto frontier allows subpar ACOs to learn from their high-value peers. For example, ACO A3835 (Connected Care of East Tennessee, LLC) was evaluated in comparison with A3791 (University Health ACO, LLC) in 2021 as its peer on the value frontier (Table 1). Both ACOs are based in Tennessee and had similar patient populations and risk scores. However, the University Health ACO was able to utilize lower input resources while achieving better quality outcomes, compared with the Connected Care ACO of East Tennessee. The DEA results suggest that A3835 had a value score of 0.69 mostly because of over-staffing by 45.8% of primary care physicians and 27% for specialists, while providing inferior patient/caregiver experience by 20.5%. Furthermore, to move to the Pareto optimal frontier, ACO A3835 also needs to reduce its annual expenditures by $389.3 per patient and increase the composite quality score for preventive health by 3.7 percentage points.

**Table 1.** Illustrative example of individual accountable care organizations with frontier peers, 2021.

	A3791 University health ACO, LLC	A3835 Connected care of East Tennessee, LLC	Gap (ratio)
Value score	1.00	0.69	
DEA inputs: clinical resources			
Health expenditure per beneficiary year	$6539.3	$6899.6	$360.3 (5.2%)
Capital expense per beneficiary year	$355	$384	$29 (7.6%)
No. of primary care physicians	129	238	109 (45.8%)
No. of physician specialists	257	393	106 (27%)
No. of other clinicians	0	0	0 (−^a^)
DEA outputs: patient outcome scores			
Patient/caregiver experience	99.1	82.2	16.9 (20.5%)
Care coordination/patient safety	100	100	0 (0%)
Preventive health	90.6	86.9	3.7 (4.3%)
At-risk population	92.5	92.5	0 (0%)
ACO characteristics			
Location (state)	TN	TN, GA, AL	
No. of assigned beneficiaries	9985	8901	
Weighted HCC risk score	0.98	0.98	

Abbreviations: ACO, accountable care organization; DEA, data envelopment analysis; HCC, Hierarchical Condition Category.

Source: Authors’ analysis of data from the Medicare Shared Savings Program public use files for 2021.

^a^Covariates are not in percent format for ease of presenting regression coefficients.

### Regression results

We used a multivariate linear regression model to estimate the relationships between ACO characteristics, SDOH variables, and health care value (Table 2). Hospital-managed ACOs that were either hospital-led or co-led with physicians exhibited 0.111-point higher value scores, on average, than their physician-led counterparts.^5^ Since hospital-managed ACOs can offer a comprehensive spectrum of care, they are more likely to ensure that patient needs are met within the ACO.^13^ This enables them to improve performance through better management of patient referrals.^20^ Our results on the role of hospital management in ACO governance and its impact on health care value were markedly different from prior research, which suggested that physician-led ACOs outperformed those managed by hospitals with respect to quality outcomes.^14,23^

**Table 2.** Estimation of accountable care organization characteristics and social determinants of health on health care value, 2014–2021.

Variable	Regression coefficient	[95% Confidence interval]
ACO characteristics		
Hospital-managed (ref: physician-led)	0.111^a^	[0.092, 0.130]
No. of years in MSSP	−0.004	[−0.023, 0.015]
Two-sided risk (ref: 1-sided risk)	0.014	[−0.006, 0.035]
Advance payment or ACO investment (ref: no participation)	−0.008	[−0.128, 0.111]
No. of assigned beneficiaries (1000’s)	0.002^a^	[0.001, 0.003]
No. of distinct participating organizations (100’s)	−0.035^b^	[−0.059, −0.011]
Weighted HCC risk score	−0.422^a^	[−0.546, −0.299]
Beneficiary concentration in ACO service counties (HHI)	0.129^b^	[0.047, 0.211]
Home health agency expenses to total health expenses^c^	−0.057	[−0.446, 0.332]
SDOH		
Economic well-being		
Unemployment rate^c^	−1.809^a^	[−2.834, −0.784]
Median household income ($10 000’s)	0.023^d^	[0.000, 0.045]
Transportation convenience		
Commute by public transportation^c^	0.442	[−0.175, 1.058]
Mean commute time (10 min)	−0.059	[−0.154, 0.035]
Healthy food consumption		
Low-income population with grocery store access^c^	3.978^b^	[1.089, 6.867]
Per capita expenses on fast food ($100’s)	−0.008	[−0.058, 0.042]
Health resource availability		
Hospital-based registered nurse to bed ratio	0.108	[−0.033, 0.249]
Observed/expected ratio of primary care checkups	0.570^b^	[0.180, 0.961]
Average distance between health care organizations (10 miles)	−0.010	[−0.023, 0.003]

Abbreviations: ACO, accountable care organization; HCC, Hierarchical Condition Category; HHI, Herfindahl-Hirschman Index; MSSP, Medicare Shared Savings Program; SDOH, social determinants of health.

Source: Authors’ analysis of data from the MSSP public use files, American Community Survey, Food Environment Atlas, Dartmouth Atlas of Healthcare, and Healthcare Information and Management Systems Society. A total of 220 ACOs in the 2012/2013 cohort are excluded because the Centers for Medicare and Medicaid Services (CMS) did not publish information on ACO service areas in 2013, leading to missing SDOH. Two more observations were removed due to missing list of participants. Thus, the model uses a sample of 3628 (94.2%) ACO-year observations (unit of analysis) that consists of 858 distinct ACOs from 2014–2021.

^a^ *P* < .001.

^b^ *P* < .01.

^c^Covariates are not in percentage format for ease of presenting regression coefficients.

^d^ *P* < .05.

ACO size had a counter-balancing effect. On one hand, an increase of 1000 ACO beneficiaries was associated with 0.002 points higher average value by allowing ACOs to spread their fixed costs across a larger patient population, thereby reducing resource utilization per beneficiary. (In separate analyses, we observed that ACO value is not significantly associated with primary care service utilization and the size, risk, and location of the patient population, suggesting that patient self-selection is not a serious concern.) On the other hand, additional distinct participating entities may offset this benefit by 0.035 points due to coordination challenges between entities. Furthermore, ACOs with riskier patient populations reported lower value since they required more clinical resources and may find it difficult to achieve quality outcomes. We also observed that a 1% increase in the concentration of beneficiary locations was associated with 0.001-point higher average value score.

#### Social determinants of health

Several SDOH factors significantly affected ACO value. Specifically, every percentage point increase in unemployment rate within the ACO service region decreased its value score by 0.018 points, while a $10 000 increase in median household income was associated with 0.023 points in higher value. Similarly, a 1% improvement in availability of healthy food options (based on grocery store access) and access to primary care was associated with increases of 0.04 points and 0.006 points in ACO value, respectively.

Other SDOH variables were not significantly associated with ACO value, possibly because these variables may be correlated with other SDOH variables, attenuating their effects. We performed Wald F tests to examine the joint significance of each SDOH domain. We observed that economic well-being factors (unemployed workforce and median household income) were jointly significant, with *P* < .001, while transportation convenience was not significant (*P* = .27). The combined factors of healthy food consumption (*P* < .05) and health resource availability (*P* < .01) were also significantly associated with ACO value. Hence, our results underscore the importance of SDOH factors, such as economic well-being, food security, and access to health resources, in determining the value of care delivered by ACOs.

We performed additional sensitivity analyses to ensure the robustness of regression results, as described in the online Appendix section F.

## Discussion

The ACO program delivered $4.3 billion in Medicare savings in 2022 alone and over $21 billion since its inception over a decade ago.^24^ Given its success and potential to improve the value of health care, CMS has set a goal to expand the ACO model to all Medicare patients by 2030.^25^ Toward this end, CMS has proposed extending accountable care coverage by recognizing the role of nurse practitioners, physician assistants, and clinical nurse specialists in delivering primary care services. It also continues to refine its benchmark methodology to encourage participation by ACOs caring for medically complex beneficiaries. Despite these endeavors to advance CMS's value-based care strategy, there remains much room for ACOs to further improve health care value. Our DEA-based approach can help ACO professionals identify inefficiencies in clinical resource utilization, patient outcomes that can be improved, and organizational factors that impact the relative value of care delivered. Our results highlight the following challenges that policymakers need to address for ACOs to achieve their full potential of high-value care.

### Dis-economies of scale

Accountable care organizations are likely to grow by increasing enrollment of Medicare beneficiaries and expanding the number of provider entities in their networks. On one hand, our study indicates that higher enrollments will allow ACOs to generate greater cost savings by spreading fixed costs across a larger number of beneficiaries. On the other hand, free-riding and coordination challenges may arise when more provider organizations join the ACO network, leading to subpar performance.^10^ Such dis-economies of scale may outweigh the benefits of size, raising risks under antitrust laws if they result in increased prices, offer fewer choices for consumers and payers, and pose challenges to improve health equity and inclusion. Our study suggests that more participating entities do not necessarily yield higher value, and ACO governance needs to strike the right balance between the number of Medicare beneficiaries served and provider entities in the network to deliver high-value care. However, CMS rules stipulate ACOs to have a minimum of 5000 beneficiaries, which favors larger organizations. This may exacerbate health disparities because larger practices already have more resources and can provide better health care.^22^ Future regulations should increase incentives for smaller entities to participate.

### Care coordination

One remedy to address dis-economies of scale is to encourage health information sharing and care coordination between participants, which not only amplifies organizational learning by sharing best practices that benefit smaller physician practices but also facilitates clinical decision marking that ensures continuity of care. The ACO governing boards can monitor participating providers and evaluate their adherence to clinical protocols, which may, in turn, improve operational transparency to quality and cost data and facilitate greater alignment among ACO participants.^21,26^ Despite these benefits, we observe that care coordination remains a significant challenge, as demonstrated by substantial slacks in this quality domain. Compared with other value-based care initiatives, the MSSP has relatively few requirements for care coordination and quality improvement.^25^ Thus, our research amplifies recent calls for CMS to further emphasize care coordination measures in their reporting of ACO performance, such as the Advancing All-Payer Health Equity Approaches and Development (AHEAD) model that CMS plans to roll out to improve care coordination, integrate mental health into overall care management, and improve health equity. Furthermore, our results also suggest that ACO managers should develop effective guidance to support care management across disparate health care providers.

### Economies of scope and patient retention

Accountable care organizations are evaluated by patient outcomes across the entire episode of care, including services delivered by providers outside their network who assume no accountability for ACO performance.^5^ This exposes ACOs to the risk of performance contamination.^20^ Therefore, ACOs should encourage broader specialization of labor to accommodate the needs of diverse patient populations, so that participating ACO providers can refer patients to providers within the same network.

Noticeably, nearly half of all ACOs in our study were led by physician groups, which have more room to grow compared with hospital-managed ACOs.^27^ On one hand, ACOs focus on avoiding costly hospital admissions, squeezing operating margins for participating hospitals to maintain capital-intensive facilities.^12^ On the other hand, hospital-managed ACOs are less likely to earn shared savings but are required to bear downside risk early.^25^ Our results suggest that future policies should encourage hospital participation in providing the entire spectrum of necessary medical care and promote incentives for physician-led ACOs to work closely with local hospitals for care management.

The positive association between beneficiary concentration and ACO value also supports such complementary partnerships. By servicing a specific region, ACOs can connect with other health care providers and coordinate care for beneficiaries due to their geographical proximity. However, in our sample of 865 distinct MSSP ACOs, 440 operated in multiple states and 143 even spanned across far-flung states. This fragmented presence may create barriers to patient coordination and retention. Our results suggest that policymakers must closely monitor the performance of these ACOs and their care delivery.

### Social determinants of health

Our research highlights the critical role of nonclinical SDOH factors on health care value. Specifically, ACOs serving socially disadvantaged areas exhibited lower value. While health care providers do not directly control their patients’ SDOH, it is important to implement policy regulations that alleviate their impact and reduce health disparities. A starting point would be to collect and document SDOH information about patients’ living conditions, such as housing stability, food security, work stress, and social support. Clinicians can implement an automated tool that allows patients to enter these data during their visits and include them in the clinical workflow.^28^ The benefits of doing so would include better prediction of adverse health events and clinical guidelines that consider these factors for diagnoses and treatment decisions.^29^

Recently, CMS launched the ACO Realizing Equity, Access, and Community Health (REACH) model to bridge health disparities in underserved communities.^25^ Our findings support this initiative and identify an array of socioeconomic characteristics that should be considered. For example, ACOs may offer necessary social services, nutritional counseling, transportation aid, and telehealth options to improve the value of care delivery. Further, the current benchmark only considers ACOs’ historical spending patterns and regional trends, which may not be adequate. Future programs should include broader SDOH in risk adjustment that can advance accountable care coverage in areas with disparities in health care access.

## Conclusion

Despite the record-breaking performance, ACOs have not achieved their full potential. We propose a novel, multidimensional measure of health care value that holistically encompasses both resource utilization and achievement of patient health outcomes, unlike prior research that focused on a single performance measure. Our value-based framework identifies the importance of ACO characteristics and social determinants and their impact on high-value patient care. Our results suggest a “skinny in scale, broad in scope” approach for ACOs to further improve resource utilization and deliver greater care value. Furthermore, to expand access to affordable care, ACOs must address the challenges of patient health equity and inclusion. For instance, ACOs can emphasize closer collaboration with local public health authorities to bridge disparities in access to care. Health policymakers can also promote the use of health information exchanges for care coordination such that ACOs have timely access to patient health data.

## Supplementary Material

qxae028_Supplementary_Data
