# Supplementary material for: Measuring value in health care: lessons from accountable care organizations
Source: Health Aff Sch. 2024 Mar 1;2(3):qxae028. doi: 10.1093/haschl/qxae028 (PMC10986292; doi:10.1093/haschl/qxae028)
Supplement: qxae028_Supplementary_Data [file qxae028_supplementary_data.zip › ACO value - Health Affairs Scholar - appendix R3 v2.docx]

**ONLINE APPENDIX**

**Section A. Data Envelopment Analysis Model Specification**

We utilized the slacks-based measure (SBM) data envelopment analysis (DEA) model to estimate healthcare value of ACOs.1 The SBM model considers the input excesses and output shortfalls, termed slacks. This allowed us to identify those ACOs that did not provide high-value care (i.e., have non-zero slacks in inputs and outputs) and increased the discrimination power.2 We used a non-oriented model, which minimizes inputs while simultaneously maximizing outputs. This specification aligns with the ACO incentive structure, as ACOs receive financial rewards based on their cost (inputs) and quality performance (outputs). Our model is specified in equation (1).

(1)

***X*** and ***Y*** represent the vectors of inputs and outputs, respectively. Index *o* indicate the focal ACO unit under evaluation. By analyzing each ACO, this fractional program of *m* inputs and *n* outputs identified a Pareto frontier, which consist of best-value performers that had zero input and output slacks, represented by and , respectively. The first two constraints and define this property as no other ACOs may outperform the frontier (achieve lower inputs and higher outputs at the same time).

The model iteratively evaluated each ACO by searching for the most similar best-value peers based on the set of inputs and outputs. The objective function (equation of ) compared the focal ACO with its benchmark peers to calculate the input and output slacks, which were then normalized by the current performance level and aggregated to derive . Thus, our DEA-based healthcare value measured the ratio at which input resources were expended to obtain quality outcomes. The derived was the value score, bounded between zero and one, with a larger score representing greater value of care delivered. We also accounted for variable returns to scale in the production function using the constraint .3

Researchers have resorted to several approaches to infer healthcare value, including stochastic frontier analysis, quadrant diagrams, weighted composite scores, and regression analysis. However, these methods are subject to significant methodological limitations, which may explain their limited use in practice. For instance, stochastic frontier estimation was predominantly developed for a single output variable and imposes strict assumptions about the functional form of the production function.4,5 While quadrant diagrams are mostly qualitative and require appropriate benchmarks for fair comparisons, the single composite score approach generally lacks standard weights to combine measures. Parametric methods, such as regressions, estimate value scores relative to the average performance, which may bias the results.6

Compared to these methods, DEA offers several advantages that address key empirical challenges, thereby serving as an appropriate approach to measure healthcare value. First, DEA considers multiple inputs and multiple outputs in the production function, allowing us to estimate the multi-dimensional nature of healthcare value. Second, DEA is a non-parametric estimation approach and does not require *a priori* specification of the underlying production function, which is especially relevant for a complex health care delivery model such as ACOs.7 Third, ACOs strive to improve cost efficiency and quality outcomes to receive the incentive payment. This makes the SBM DEA model particularly appealing to quantify the value of healthcare provided by ACOs, since it is consistent with the ACO objective function to minimize input sets while simultaneously maximizing output variables. Lastly, by identifying peer ACOs as the comparison benchmark, DEA allows us to identify potential improvement opportunities, thereby offering useful insights.

**Section B. Illustration of Data Envelopment Analysis**

In this section, we utilize an illustrative example of one input resource and one quality output to explain the intuition of slacks-based measure (SBM) data envelopment analysis (DEA). We consider seven ACOs – A, B, C, D, E, O, and P – in the coordinate system of input resource ***X*** and quality output ***Y*** (Figure B1). ACOs that are located in the upper left quadrant exhibit greater healthcare value because they use fewer input to deliver better quality output. DEA recognizes the unique production set of each ACO. It identifies a Pareto frontier that consists of best-value ACOs, i.e., A, B, C, D, and E (value score of 1). This means that no other ACO can achieve the same level of output with less input (or use the same amount of input to generate better output).

ACO O is not on the Pareto frontier because it can improve its value score by moving left (reducing input consumption) and/or upward (increasing quality output). DEA considers ABCDE as the reference group and pairs the closest one with O based on the input and output set, i.e., ACO D. In other words, ACO D serves as the target for O to benchmark on. Compared with ACO D, O has both input excess and output shortfall, as indicated by red arrows, that measure the gap between current and optimal performance levels (DEA slacks).

The SBM DEA model addresses the measurement units by normalizing each slack as a ratio of its current level (input excess/current input and output shortfall/current output). These ratios measure the extent of improvement that ACO O needs to undertake in the input and output to reach its target D (named gap ratio in Figure 2 of the manuscript). The SBM DEA model calculates the value score for ACO O as (1 – input gap ratio)/(1 + output gap ratio).

Intuitively, if ACO P is also paired with D but has a higher input and lower output, it will exhibit larger slacks, and therefore a lower value score. Thus, in general, the SBM DEA model identifies the Pareto frontier with the best-value ACOs, benchmarks off-frontier ACOs with the closest best performers, and calculates value scores ranging from 0 to 1 with a higher number indicating greater value of care delivery.

**Figure B1. Illustrative example of slacks-based measure data envelopment analysis**

**P**

Current

Output

Current Input

Output shortfall

(output slack)

Input excess (input slack)

**O**

**E**

**D**

**C**

**B**

**A**

Pareto Frontier

Input Resource *X*

Output

Quality *Y*

High healthcare value

Low healthcare value

**Source:** Authors develop the scenario based on the examples in Cooper et al.2 and Tone et al.8

**Section C. Input and Output Variables in Data Envelopment Analysis**

We utilized the healthcare literature on ACOs to determine input and output variables in our data envelopment analysis (DEA).9–11 Table C1 presents the mean and 95th percentile interval of DEA inputs and outputs. The input set contained major categories of clinical resources, including operating expenses, capital investments, and labor. First, operating expenses measured the lump sum cost incurred to provide patient care during the entire episode of performance year. CMS calculates this cost by annualizing, truncating, and weighting the expenditures based on each Medicare enrollment type. This ensures that costs are adjusted for the months of Medicare eligibility, including new enrollees during the year, and accounts for outliers. Second, we used the expenses on durable medical equipment as a proxy for capital investment. This expenditure is identified by claim codes and adjusted for Medicare enrollment type. Third, we measured labor inputs separately based on the numbers of primary care physicians, specialists, and other clinicians (nurse practitioners, physician assistants, and clinical nurse specialists) who reassigned billing rights to an ACO participant during the performance year. To avoid double-counting input resources in the DEA model, we excluded the expenditures for durable medical equipment, physicians, and other suppliers from the operating expenses. We also normalized input variables to a beneficiary-year basis to account for scale effects.

In terms of DEA outputs, we considered four domains of quality measures: patient/caregiver experience, care coordination/patient safety, preventive health, and at-risk population. CMS evaluated ACO quality performance using a pre-determined set of over 30 individual measures that span a wide spectrum of clinical practices. The active measures in use differed by year. These measures were divided into the above four domains and evaluated under the purview of pay-for-reporting (P4R) or pay-for-performance (P4P), depending on the performance year. Table C2 summarizes the active measures in each performance year, a brief definition of each measure, and their categorization into the four quality domains.

Under the P4R method, ACOs receive full credit as long as they completely and accurately report quality data. However, the P4R approach may overestimate the true ACO value.12 For instance, the overall quality for MSSP was exceptional in 2013 as 198 out of 203 ACOs achieved a 100% quality score under the P4R method, while quality performance substantially dropped the next year when P4P was phased in. Hence, we only considered P4P measures in constructing DEA outputs as they are more representative of quality outcomes.

Since the P4P measures are scored using different scales and some may convey reverse meaning (e.g., readmission rate), we followed the approach used by CMS to convert them to a sliding scale between zero and two using their respective benchmarks. The quality points for each domain were then aggregated and normalized to a zero-to-one scale. A larger value indicated better patient outcomes.

**Table C1. Summary statistics of data envelopment analysis (DEA) input and output variables, 2013 – 2021**

|  | **Mean** | **95th Percentile Interval** | |
| --- | --- | --- | --- |
| **DEA Input: Clinical Resources** |  |  |  |
| Health expenditure per beneficiary year ($) | 7031.28 | [1835.70, | 12798.89] |
| Capital expense per beneficiary year ($) | 277.26 | [168, | 431] |
| No. of primary care physicians | 224.28 | [19, | 1061] |
| No. of physician specialists | 407.72 | [1, | 2317] |
| No. of other clinicians | 169.74 | [0, | 1037] |
| **DEA Output: Patient Outcomes** |  |  |  |
| P4P quality score for patient/caregiver experience domain (%) | 81.64 | [46.56, | 97.50] |
| P4P quality score for care coordination/patient safety domain (%) | 72.98 | [23.33, | 100.00] |
| P4P quality score for preventive health domain (%) | 80.78 | [46.88, | 96.25] |
| P4P quality score for at-risk population domain (%) | 83.06 | [40.00, | 97.50] |

**Source**: Authors’ analysis of data from the Medicare Shared Savings Program public use files from 2013 to 2021.

**Table C2. Accountable care organizations quality measures by year, 2013 – 2021**

| **Individual  Measure** | **Description** | **2013** | **2014** | **2015** | **2016** | **2017** | **2018** | **2019** | **2020a** | **2021b** |
| --- | --- | --- | --- | --- | --- | --- | --- | --- | --- | --- |
| ***Domain: Patient/Caregiver Experience*** | | | | | | | | | | | |
| ACO 1 | Getting Timely Care, Appointments, and Information | ✓ | ✓ | ✓ | ✓ | ✓ | ✓ | ✓ |  | ✓ |
| ACO 2 | How Well Your Doctors Communicate | ✓ | ✓ | ✓ | ✓ | ✓ | ✓ | ✓ |  | ✓ |
| ACO 3 | Patients’ Rating of Doctor | ✓ | ✓ | ✓ | ✓ | ✓ | ✓ | ✓ |  | ✓ |
| ACO 4 | Access to Specialists | ✓ | ✓ | ✓ | ✓ | ✓ | ✓ | ✓ |  |  |
| ACO 5 | Health Promotion and Education | ✓ | ✓ | ✓ | ✓ | ✓ | ✓ | ✓ |  | ✓ |
| ACO 6 | Shared Decision Making | ✓ | ✓ | ✓ | ✓ | ✓ | ✓ | ✓ |  | ✓ |
| ACO 7 | Health Status/Functional Status | ✓ | ✓ | ✓ | ✓ | ✓ | ✓ | ✓ |  |  |
| ACO 34 | Stewardship of Patient Resources |  |  | ✓ | ✓ | ✓ | ✓ | ✓ |  | ✓ |
| ACO 45 | Courteous and Helpful Office Staff |  |  |  |  |  |  | ✓ |  | ✓ |
| ACO 46 | Care Coordination |  |  |  |  |  |  | ✓ |  | ✓ |
| ***Domain: Care Coordination/Patient Safety*** | | | | | | | | | | | |
| ACO 8 | Risk Standardized, All Condition Readmissions | ✓ | ✓ | ✓ | ✓ | ✓ | ✓ | ✓ | ✓ |  |
| ACO 9 | ASC Admissions: COPD or Asthma in Older Adults | ✓ | ✓ | ✓ | ✓ |  |  |  |  |  |
| ACO 10 | ASC Admission: Heart Failure | ✓ | ✓ | ✓ | ✓ |  |  |  |  |  |
| ACO 11 | Percent of PCPs who Qualified for EHR Incentive Payment | ✓ | ✓ | ✓ | ✓ | ✓ | ✓ |  |  |  |
| ACO 12 | Medication Reconciliation | ✓ | ✓ |  |  | ✓ | ✓ |  |  |  |
| ACO 13 | Falls: Screening for Fall Risk | ✓ | ✓ | ✓ | ✓ | ✓ | ✓ | ✓ | ✓ | ✓ |
| ACO 35 | Skilled Nursing Facility 30-Day All-Cause Readmission Measure |  |  | ✓ | ✓ | ✓ | ✓ |  |  |  |
| ACO 36 | All-Cause Unplanned Admissions for Patients with Diabetes |  |  | ✓ | ✓ | ✓ | ✓ |  |  |  |
| ACO 37 | All-Cause Unplanned Admissions for Patients with Heart Failure |  |  | ✓ | ✓ | ✓ | ✓ |  |  |  |
| ACO 38 | All-Cause Unplanned Admissions for Patients with Multiple Chronic Conditions |  |  | ✓ | ✓ | ✓ | ✓ | ✓ | ✓ | ✓ |
| ACO 39 | Documentation of Current Medications in the Medical Record |  |  | ✓ | ✓ |  |  |  |  |  |
| ACO 43 | Ambulatory Sensitive Condition Acute Composite |  |  |  |  | ✓ | ✓ | ✓ | ✓ |  |
| ACO 44 | Use of Imaging Studies for Low Back Pain |  |  |  |  | ✓ | ✓ |  |  |  |
| Measure 479 | Hospital-Wide 30-Day All-Cause Unplanned Readmission Rate |  |  |  |  |  |  |  |  | ✓ |
| ***Domain: Preventive Health*** | | | | | | | | | | | |
| ACO 14 | Influenza Immunization | ✓ | ✓ | ✓ | ✓ | ✓ | ✓ | ✓ | ✓ | ✓ |
| ACO 15 | Pneumococcal Vaccination | ✓ | ✓ | ✓ | ✓ | ✓ | ✓ |  |  |  |
| ACO 16 | Adult Weight Screening and Follow-up | ✓ | ✓ | ✓ | ✓ | ✓ | ✓ |  |  |  |
| ACO 17 | Tobacco Use Assessment and Cessation Intervention | ✓ | ✓ | ✓ | ✓ | ✓ | ✓ | ✓ | ✓ | ✓ |
| ACO 18 | Depression Screening | ✓ | ✓ | ✓ | ✓ | ✓ | ✓ | ✓ | ✓ | ✓ |
| ACO 19 | Colorectal Cancer Screening | ✓ | ✓ | ✓ | ✓ | ✓ | ✓ | ✓ | ✓ | ✓ |
| ACO 20 | Breast Cancer Screening | ✓ | ✓ | ✓ | ✓ | ✓ | ✓ | ✓ | ✓ | ✓ |
| ACO 21 | Screening for High Blood Pressure and Follow-Up Documented | ✓ | ✓ | ✓ | ✓ |  |  |  |  |  |
| ACO 42 | Statin Therapy for the Prevention and Treatment of Cardiovascular Disease |  |  |  | ✓ | ✓ | ✓ | ✓ | ✓ | ✓ |
| ***Domain: At-Risk Population*** | | | | | | | | | | | |
| Diabetes Comp. | Composite Measures for Diabetes Patients | ✓ | ✓ | ✓ | ✓ | ✓ | ✓ |  |  |  |
| ACO 27 | Diabetes Mellitus: Hemoglobin A1c Poor Control | ✓ | ✓ |  |  |  |  | ✓ | ✓ | ✓ |
| ACO 28 | Hypertension: Controlling High Blood Pressure | ✓ | ✓ | ✓ | ✓ | ✓ | ✓ | ✓ | ✓ | ✓ |
| ACO 29 | Ischemic Vascular Disease: Complete Lipid Panel and LDL Control | ✓ | ✓ |  |  |  |  |  |  |  |
| ACO 30 | Ischemic Vascular Disease: Use of Aspirin or Another Antithrombotic | ✓ | ✓ | ✓ | ✓ | ✓ | ✓ |  |  |  |
| ACO 31 | Heart Failure: Beta-Blocker Therapy for Left Ventricular Systolic Dysfunction | ✓ | ✓ | ✓ | ✓ |  |  |  |  |  |
| CAD Comp. | Composite Measures for Coronary Artery Disease Patients | ✓ | ✓ |  |  |  |  |  |  |  |
| ACO 33 | ACE Inhibitor or ARB Therapy |  |  | ✓ | ✓ |  |  |  |  |  |
| ACO 40 | Depression Remission at Twelve Months |  |  | ✓ | ✓ | ✓ | ✓ | ✓ | ✓ | ✓ |

**Source**: Authors’ summary of quality benchmark documentation of the Medicare Shared Savings Program from 2013 to 2021.

**Notes**: This table presents the active individual quality measures in each performance year and their categorization into four quality domains. A checkmark indicates the quality measure was active (i.e., evaluated by CMS using either pay-for-reporting or pay-for-performance method) in a particular year.

**a** In 2020, CMS waived the requirement to file the Consumer Assessment of Healthcare Providers and Systems (CAHPS) for ACOs survey, and all ACOs automatically received full credit for the patient/caregiver experience measures. Hence, all measures under this domain are unchecked.

**b** Since 2021, CMS undertook major structural changes to the quality reporting system, to better align with the Quality Payment Program (QPP) Merit-Based Incentive Payment System (MIPS). The revamped version defined the new reporting mechanism, quality measures, and scoring rules. The year 2021 was the transition period where ACOs could choose the traditional Web Interface Measures or the new clinical quality measures (CQMs). We report the quality measures of the former approach because most ACOs used this option, which includes the required measures of the latter approach. We mapped the newly defined quality measures with the previous ones based on their description.

**Section D. Independent Variables in Regression Analysis**

Based on the extant literature, we considered several ACO-level factors that may affect ACO operations and performance.13,14 It has been reported that ACOs with hospital participants are able to better utilize IT systems to manage inpatient care and emergency visits, whereas those operated solely by physicians focus on ambulatory services to reduce costly admissions.15 We constructed a binary variable (*hospital-managed*) to indicate if an ACO included hospital participants (hospital-led or co-led with physician groups).16 To account for potential learning effects, we defined the number of years an ACO has enrolled in the MSSP (*No. of years in MSSP*).17 The selection of risk model determines whether an ACO is subject to downward financial risk (i.e., penalty). We included a binary indicator, *two-sided risk*, that is equal to one if an ACO participated in the two-sided risk model (i.e., both reward and penalty), and zero otherwise.14 Rural ACOs or those serving underserved areas can opt to participate in the Advance Payment Model or ACO Investment Model, where they received financial support to invest in infrastructure for care coordination.18 We measured this using a binary indicator *advance payment or ACO investment*.

Furthermore, we utilized two measures for ACO size – the *number of assigned beneficiaries* and the *number of distinct participating organizations*, calculated using their taxpayer IDs.19 Since riskier patients consumed more resources, but exhibited lower health outcomes, we controlled for the severity of the patient population. We defined the variable, *weighted HCC risk score*, as the average Hierarchical Condition Category (HCC) risk score, weighted by the number of patients for each type (end-stage renal disease, disabled, aged/dual, and aged/non-dual). A higher number indicates a more severe patient mix served. Further, ACOs may potentially serve a large geographical region, which can create barriers in coordinating patient care. Therefore, we constructed a Herfindahl-Hirschman Index (HHI) using the beneficiary size in each service county of ACO operation.19 The resulting variable *beneficiary concentration* is continuous from zero to one. A larger number suggests a more concentrated ACO service area. Last, we calculated a ratio of *home health agency expense to total health expense* because post-acute care provides an avenue for patient recovery after discharge and may prevent expensive hospital readmissions.20

Although previous ACO studies have not examined SDOH factors, their findings imply that ACO performance may be driven by the regional market.12,21 We followed the extant literature to explore the effect of regional factors.22–26 Note that these determinants were not directly related to ACO patients, but instead, based on their geographic residence. Specifically, we used *unemployment rate* and *median household income* to proxy for the economic well-being of the counties that an ACO served. With respect to transportation convenience, we considered the percentage of workforce who *commute by public transportation* and the *average commute time* needed to work. We posited that greater use of public transportation and less commute time are proxies for the quality of transportation infrastructure.

In addition, we measured the propensity of healthy food consumption using two variables: the percentage of *low-income population with grocery store access* and *per capita expenses on fast food*. Access to grocery stores and low fast-food expenses may indicate a patient population with healthier lifestyles. Lastly, we considered three levels of health resources. At the hospital level, we calculated the total number of hospital-based registered nurses in a county, divided by the sum of hospital beds. For ambulatory settings, we constructed a composite score for Medicare patients who received annual primary checkups (e.g., eye exams for diabetes patients and mammograms for females aged above 67). Further, using the latitude and longitude location of healthcare organizations, we calculated the average distance between medical facilities which is an indicator of the density of medical resources in a region. These SDOH factors were initially collected at the county level and then aggregated to ACO-year units using the number of beneficiaries in each county as the weight. Table D1 presents the mean and 95th percentile interval of all independent variables used in the regression analysis.

**Table D1. Summary statistics of accountable care organization (ACO) characteristics, and social determinants of health, 2014 – 2021**

|  | **Mean** | **95th Percentile Interval** | |
| --- | --- | --- | --- |
| **ACO Characteristics** |  |  |  |
| ACO leadership (%) |  |  |  |
| Physician-led (ref) | 47.52 | [0, | 1] |
| Hospital-managed (Hospital- or joint-led) | 52.48 | [0, | 1] |
| No. of years in MSSP | 2.57 | [0, | 7.5] |
| Risk model (%) |  |  |  |
| One-sided risk (ref) | 82.64 | [0, | 1] |
| Two-sided risk | 17.35 | [0, | 1] |
| Advance Payment or ACO Investment Model (%) | 9.50 | [0, | 1] |
| No. of assigned beneficiaries (1,000s) | 19.33 | [4.78, | 74.15] |
| No. of distinct participating organizations (100s) | 0.36 | [0.01, | 1.75] |
| Weighted HCC risk score | 1.03 | [0.86, | 1.26] |
| Beneficiary concentration in ACO service counties (HHI) | 0.34 | [0.05, | 0.88] |
| Home health agency expense to total health expense (%) | 5.46 | [2.04, | 11.60] |
| **Social Determinants of Health** |  |  |  |
| Economic well-being |  |  |  |
| Unemployment rate (%) | 6.60 | [3.65, | 11.24] |
| Median household income ($10,000s) | 6.09 | [4.02, | 9.74] |
| Transportation convenience |  |  |  |
| Commute by public transportation (%) | 3.77 | [0.21, | 25.09] |
| Mean commute time (10 mins) | 2.59 | [1.84, | 3.60] |
| Healthy food consumption |  |  |  |
| Low-income population with grocery store access (%) | 2.03 | [0.29, | 4.39] |
| Per capita expenses on fast food ($100s) | 5.81 | [4.27, | 7.29] |
| Health resource availability |  |  |  |
| Hospital-based registered nurse to bed ratio | 2.07 | [1.53, | 2.69] |
| Observed/expected ratio of primary care checkups | 1.01 | [0.92, | 1.10] |
| Average distance between healthcare organizations (10 miles) | 1.08 | [0.26, | 3.26] |

**Source**: Authors’ analysis of data from the Medicare Shared Savings Program (MSSP) public use files, American Community Survey, Food Environment Atlas, Dartmouth Atlas of Healthcare, and Healthcare Information and Management Systems Society.

**Notes**: A total of 220 ACOs in the 2012/2013 cohort are excluded because the Centers for Medicare and Medicaid Services (CMS) did not publish information on ACO service county in 2013, leading to missing values for social determinants. Two more observations are removed due to missing participant lists. Summary statistics are based on a sample of 3,628 ACO-year observations (858 distinct ACOs from 2014 to 2021). HCC is Hierarchical Condition Category. HHI is Herfindahl-Hirschman Index.

**Section E. Multivariate Regression Model**

We used a multivariate linear regression model to examine factors associated with healthcare value in accountable care organizations (ACOs). The model is specified in equation (2).

(2)

where the dependent variable is the healthcare value score based on data envelopment analysis for ACO *i* in year *t*. *ACOChar* is a vector of time-varying factors that measures organizational characteristics at the ACO level and *SDOH* is a list of variables for the social determinants of health (SDOH) in the counties that ACO served. A detailed description of these variables are presented in Section D. represents ACO-specific fixed effects to account for time-invariant ACO heterogeneity. represents year fixed effects to account for any changes in program regulations and macroeconomic environment that affect all ACOs. is the idiosyncratic error term that captures unobserved random factors that may affect healthcare value.

The estimation results are reported in Table 4 in the manuscript. Table E1 presents complementary results, including standard errors, R-squared, and F statistics.

**Table E1. Complementary results for the effect of accountable care organization (ACO) characteristics and social determinants of health (SDOH) on healthcare value, 2014 – 2021**

| **Independent Variables** | **Regression**  **Coefficients** | **Standard**  **Errors** |
| --- | --- | --- |
|  |  |  |
| **ACO Characteristics** |  |  |
| Hospital-managed (ref: physician-led) | 0.111**** | (0.010) |
| No. of years in MSSP | -0.004 | (0.009) |
| Two-sided risk (ref: one-sided risk) | 0.014 | (0.011) |
| Advance Payment or ACO Investment (ref: no participation) | -0.008 | (0.061) |
| No. of assigned beneficiaries (1,000s) | 0.002**** | (0.0004) |
| No. of distinct participating organizations (100s) | -0.035*** | (0.012) |
| Weighted HCC risk score | -0.422**** | (0.063) |
| Beneficiary concentration in ACO service counties (HHI) | 0.129*** | (0.042) |
| Home health agency expense to total health expensea | -0.057 | (0.198) |
| **Social Determinants of Health** |  |  |
| Economic well-being |  |  |
| Unemployment ratea | -1.809**** | (0.523) |
| Median household income ($10,000s) | 0.023** | (0.011) |
| Transportation convenience |  |  |
| Commute by public transportationa | 0.442 | (0.314) |
| Mean commute time (10 mins) | -0.059 | (0.048) |
| Healthy food consumption |  |  |
| Low-income population with grocery store accessa | 3.978*** | (1.473) |
| Per capita expenses on fast food ($100s) | -0.008 | (0.026) |
| Health resource availability |  |  |
| Hospital-based registered nurse to bed ratio | 0.108 | (0.072) |
| Observed/expected ratio of primary care checkups | 0.570*** | (0.199) |
| Average distance between healthcare organizations (10 miles) | -0.010 | (0.007) |
| Constant | 0.383 | (0.306) |
|  |  |  |
| Number of ACO-year observations | 3,628 | |
| Number of distinct ACOs | 858 | |
| R-squared | 0.108 | |
| F statistics | 10.08 | |

**Source**: Authors’ analysis of data from the Medicare Shared Savings Program (MSSP) public use files, American Community Survey, Food Environment Atlas, Dartmouth Atlas of Healthcare, and Healthcare Information and Management Systems Society.

**Notes**: This table shows the complementary results of Table 4, including standard errors (in parentheses), R-squared, and F statistics. All other notes remain the same.

**p < 0.05 ***p < 0.01 ****p < 0.001.

**Section F. Sensitivity Regression Analyses**

We performed several sensitivity analyses to ensure the robustness of our results. First, we compared organizational characteristics and SDOH variables between high- and low-value ACOs to mitigate potential outlier effects. We defined a binary variable to indicate high-value ACOs and regressed it on ACO characteristics and SDOH variables. We utilized a linear probability model since logit regression with fixed effects may lead to incidental parameter bias. The results in Table F1 suggest that high-value ACOs were more likely to be managed by hospitals, include fewer entities, have denser patient populations, and located in economically prosperous areas with greater access to health resources.

Second, we adopted a window analysis approach to account for temporal trends in ACO value.2 We ran DEA on a rolling window of two years where the healthcare value of the focal ACO was evaluated against its own performance in a different year as well as compared to other ACOs. The resulting value score was used as the dependent variable to re-estimate the multivariate regression. Table F2 presents the estimation results. We observed qualitatively similar results that ACOs with higher value scores were more likely to be managed by hospitals, comprise fewer entities, and served a large group of beneficiaries. Furthermore, we observed that the number of years of MSSP enrollment was negatively associated with healthcare value.

In separate analyses, we found that longer MSSP enrollment was associated with higher level of health expenditures, likely due to the rebasing mechanism of cost benchmarks that diminishes ACO incentives to reduce costs over time.12,27 While ACO experience also improves quality outcomes, its magnitude is equal to the increase in costs, leading to a reduction in healthcare value. Finally, SDOH factors, including economic well-being, transportation convenience, and healthy food consumption, were also significantly associated with ACO value.

Third, the ACO quality measure # 11 (see Table C2), which measures the percentage of primary care physicians who received electronic health record (EHR) incentive payments, represents EHR use and may be considered as an input resource in the production of patient outcomes. Hence, we excluded this measure when calculating the quality score for care coordination (i.e., the quality domain of this measure) and added it to the set of DEA inputs. We used the new value score as the dependent variable and reran the regression model. Table F3 reports the regression results where the coefficients are qualitatively consistent, although some factors are less significant. In a separate analysis, we also removed the care coordination measure as a DEA output and observed largely consistent regression results.

Fourth, it is important that the measure of healthcare value takes into account the heterogeneity of the patient population. Our regression analysis controlled the number, risk score, and geographical location of assigned beneficiaries. We further addressed this concern by implementing a stratified DEA method. Specifically, we split ACOs evenly into three groups for each year based on the size of the patient population. We performed a stratified DEA within each group where ACOs are comparable in size. Using the same regression model specification, we report the estimation results in Table F4, which yields consistent findings.

Furthermore, we also accounted for changes in the number of ACO beneficiaries by including the relative change in ACO size as an additional DEA output. From a policy perspective, this specification may be plausible as CMS not only expects ACOs to improve the value of care delivery but also expand accountable care coverage. Considering the potential patient self-selection due to ACO performance, we calculated a ratio measure of the change in ACO patient population instead of using the raw patient count. A value larger than one indicates an increase in ACO size, likely attributed to improvements in ACO performance. Our regression results based on relative changes in ACO beneficiary population are consistent with the main results reported in the paper (not reported).

A possible concern may be that ACO leadership taxonomy (hospital-managed vs. physician-led) and risk tracks (one-sided vs. two-sided) do not have enough variation after controlling for fixed effects. In our sample of 865 distinct ACOs, 135 ACOs (826 ACO-year observations) changed leadership type, among which 52 switched from physician-led to hospital-managed, 91 changed in the opposite direction, and 6 had multiple leadership changes. We also observed that 158 ACOs (1110 observations) switched from one-sided to two-sided risk tracks. Hence, hospital-managed (vs. physician-led) and two-sided risk (vs. one-sided risk) are time-variant variables. To further alleviate this concern, we performed a subsample regression using ACOs that changed leadership or switched risk tracks. Since we are interested in the effect of leadership taxonomy and risk tracks, we only report their coefficients in Table F5 for brevity, although other variables were also included. Consistent with our main findings, hospital-managed ACOs that were either hospital-led or co-led with physicians exhibited a higher average value score compared to their physician-led counterparts. While switching to two-sided risk tracks is associated with a higher ACO value, the effect is marginally significant (p-value = 0.072).

Finally, since SDOH factors within one category may be correlated with each other, we examined the pairwise correlations (not reported) and observed that the highest correlation occurred between public transportation and commute time (0.68), as expected. The other correlation coefficients were generally below the acceptable threshold of 0.8. We also calculated the variance inflation factor (VIF) for each variable and found that VIFs were all lower than the cutoff of five. Collectively, our tests suggest that multicollinearity is not a serious concern. We also dropped variables with high VIF (i.e., household income and commute time with VIF=4.77), reran the regression model, and present the estimated results for SDOH factors in Table F6, which lead to similar conclusions.

**Table F1. Linear probability regression results for differences between high- and low-value accountable care organizations (ACOs), 2014 – 2021**

| **Variable** | **Regression Coefficient** | **95% Confidence Interval** | |
| --- | --- | --- | --- |
|  |  |  |  |
| **ACO Characteristics** |  |  |  |
| Hospital-managed (ref: physician-led) | 0.247**** | [0.155, | 0.339] |
| No. of years in MSSP | -0.054 | [-0.154, | 0.047] |
| Two-sided risk (ref: one-sided risk) | 0.011 | [-0.094, | 0.117] |
| Advance Payment or ACO Investment (ref: no participation) | -0.126 | [-0.602, | 0.349] |
| No. of assigned beneficiaries (1,000s) | 0.004 | [-0.001, | 0.008] |
| No. of distinct participating organizations (100s) | -0.151*** | [-0.249, | -0.053] |
| Weighted HCC risk score | -0.334 | [-0.816, | 0.147] |
| Beneficiary concentration in ACO service counties (HHI) | 0.493** | [0.037, | 0.949] |
| Home health agency expense to total health expensea | -0.513 | [-2.141, | 1.115] |
| **Social Determinants of Health** |  |  |  |
| Economic well-being |  |  |  |
| Unemployment ratea | -3.053 | [-8.523, | 2.416] |
| Median household income ($10,000s) | 0.218**** | [0.125, | 0.311] |
| Transportation convenience |  |  |  |
| Commute by public transportationa | -0.593 | [-3.073, | 1.887] |
| Mean commute time (10 mins) | -0.310 | [-0.800, | 0.180] |
| Healthy food consumption |  |  |  |
| Low-income population with grocery store accessa | -1.283 | [-14.973, | 12.406] |
| Per capita expenses on fast food ($100s) | -0.264 | [-0.637, | 0.109] |
| Health resource availability |  |  |  |
| Hospital-based registered nurse to bed ratio | -0.202 | [-0.994, | 0.590] |
| Observed/expected ratio of primary care checkups | -0.142 | [-2.071, | 1.786] |
| Average distance between healthcare organizations (10 miles) | -0.221**** | [-0.342, | -0.099] |

**Source**: Authors’ analysis of data from the Medicare Shared Savings Program (MSSP) public use files, American Community Survey, Food Environment Atlas, Dartmouth Atlas of Healthcare, and Healthcare Information and Management Systems Society.

**Notes**: The model uses a sample of 522 ACOs from 2014 – 2021 (1,119 ACO-year observations) that do not have missing covariates. R-squared equals 0.173 and F statistics equals 3.59. Joint significance tests are performed to examine each SDOH domain. The economic well-being factors are jointly significant (p<0.001). The transportation convenience factors and healthy food consumption factors are not jointly significant with p-values equal to 0.27 and 0.37, respectively. The health resource availability factors are jointly significant (p<0.01). HCC is Hierarchical Condition Category. HHI is Herfindahl-Hirschman Index.

aCovariates are not in percent format for the ease of presenting regression coefficients.

**p < 0.05 ***p < 0.01 ****p < 0.001.

**Table F2. Regression results for healthcare value based on window analysis, 2014 – 2021**

| **Variable** | **Regression Coefficient** | **95% Confidence Interval** | |
| --- | --- | --- | --- |
|  |  |  |  |
| **ACO Characteristics** |  |  |  |
| Hospital-managed (ref: physician-led) | 0.084**** | [0.069, | 0.099] |
| No. of years in MSSP | -0.030**** | [-0.045, | -0.016] |
| Two-sided risk (ref: one-sided risk) | -0.007 | [-0.023, | 0.009] |
| Advance Payment or ACO Investment (ref: no participation) | -0.040 | [-0.132, | 0.053] |
| No. of assigned beneficiaries (1,000s) | 0.001** | [0.000, | 0.001] |
| No. of distinct participating organizations (100s) | -0.021** | [-0.039, | -0.002] |
| Weighted HCC risk score | -0.376**** | [-0.471, | -0.280] |
| Beneficiary concentration in ACO service counties (HHI) | 0.005 | [-0.059, | 0.068] |
| Home health agency expense to total health expensea | 0.375** | [0.073, | 0.677] |
| **Social Determinants of Health** |  |  |  |
| Economic well-being |  |  |  |
| Unemployment ratea | -0.690 | [-1.485, | 0.105] |
| Median household income ($10,000s) | 0.028*** | [0.011, | 0.046] |
| Transportation convenience |  |  |  |
| Commute by public transportationa | 0.682*** | [0.204, | 1.160] |
| Mean commute time (10 mins) | -0.089** | [-0.162, | -0.015] |
| Healthy food consumption |  |  |  |
| Low-income population with grocery store accessa | 3.951**** | [1.710, | 6.193] |
| Per capita expenses on fast food ($100s) | 0.008 | [-0.030, | 0.047] |
| Health resource availability |  |  |  |
| Hospital-based registered nurse to bed ratio | 0.074 | [-0.035, | 0.183] |
| Observed/expected ratio of primary care checkups | 0.167 | [-0.135, | 0.470] |
| Average distance between healthcare organizations (10 miles) | 0.002 | [-0.008, | 0.013] |

**Source**: Authors’ analysis of data from the Medicare Shared Savings Program (MSSP) public use files, American Community Survey, Food Environment Atlas, Dartmouth Atlas of Healthcare, and Healthcare Information and Management Systems Society.

**Notes**: The model uses a full sample of 858 ACOs from 2014 to 2021 (3,625 ACO-year observations) that do not have missing covariates. R-squared equals 0.216 and F statistics equals 22.78. Joint significance tests are performed to examine each SDOH domain. The economic well-being factors are jointly significant (p<0.001), while the transportation convenience factors and healthy food consumption factors are also jointly significant (p<0.01). The health resource availability factors are not jointly significant (p=0.37). HCC is Hierarchical Condition Category. HHI is Herfindahl-Hirschman Index.

aCovariates are not in percent format for the ease of presenting regression coefficients.

**p < 0.05 ***p < 0.01 ****p < 0.001.

**Table F3. Regression results using alternate data envelopment analysis (DEA) inputs, 2014 – 2018**

| **Variable** | **Regression Coefficient** | **95% Confidence Interval** | |
| --- | --- | --- | --- |
|  |  |  |  |
| **ACO Characteristics** |  |  |  |
| Hospital-managed (ref: physician-led) | 0.105**** | [0.076, | 0.133] |
| No. of years in MSSP | -0.016** | [-0.030, | -0.001] |
| Two-sided risk (ref: one-sided risk) | 0.009 | [-0.027, | 0.044] |
| Advance Payment or ACO Investment (ref: no participation) | -0.012 | [-0.129, | 0.105] |
| No. of assigned beneficiaries (1,000s) | 0.001** | [0.000, | 0.002] |
| No. of distinct participating organizations (100s) | -0.039*** | [-0.068, | -0.010] |
| Weighted HCC risk score | -0.255*** | [-0.437, | -0.072] |
| Beneficiary concentration in ACO service counties (HHI) | -0.054 | [-0.187, | 0.080] |
| Home health agency expense to total health expensea | -0.706 | [-1.542, | 0.130] |
| **Social Determinants of Health** |  |  |  |
| Economic well-being |  |  |  |
| Unemployment ratea | -0.752 | [-2.261, | 0.757] |
| Median household income ($10,000s) | -0.010 | [-0.057, | 0.038] |
| Transportation convenience |  |  |  |
| Commute by public transportationa | 0.137 | [-0.972, | 1.246] |
| Mean commute time (10 mins) | -0.057 | [-0.210, | 0.096] |
| Healthy food consumption |  |  |  |
| Low-income population with grocery store accessa | -0.965 | [-6.086, | 4.157] |
| Per capita expenses on fast food ($100s) | -1.708** | [-3.154, | -0.261] |
| Health resource availability |  |  |  |
| Hospital-based registered nurse to bed ratio | 0.152 | [-0.102, | 0.406] |
| Observed/expected ratio of primary care checkups | 0.474 | [-0.070, | 1.019] |
| Average distance between healthcare organizations (10 miles) | -0.006 | [-0.019, | 0.008] |

**Source**: Authors’ analysis of data from the Medicare Shared Savings Program (MSSP) public use files, American Community Survey, Food Environment Atlas, Dartmouth Atlas of Healthcare, and Healthcare Information and Management Systems Society.

**Notes**: Because CMS removed the EHR use variable from the ACO quality measures since 2019, this analysis uses a subsample of 2,158 ACO-year observations from 2014 to 2021. R-squared equals 0.092 and F statistics equals 4.67. Joint significance tests are performed to examine each SDOH domain. The economic well-being (p-value=0.60), transportation convenience (p-value=0.77), and health resource availability (p-value=0.19) are not significant, while the healthy food consumption factors are marginally significant (p-value=0.068). HCC is Hierarchical Condition Category. HHI is Herfindahl-Hirschman Index.

aCovariates are not in percent format for the ease of presenting regression coefficients.

**p < 0.05 ***p < 0.01 ****p < 0.001.

**Table F4. Regression results using stratified data envelopment analysis based on patient population, 2014 – 2021**

| **Variable** | **Regression Coefficient** | **95% Confidence Interval** | |
| --- | --- | --- | --- |
|  |  |  |  |
| **ACO Characteristics** |  |  |  |
| Hospital-managed (ref: physician-led) | 0.157**** | [0.125, | 0.188] |
| No. of years in MSSP | -0.015** | [-0.028, | -0.003] |
| Two-sided risk (ref: one-sided risk) | -0.021 | [-0.056, | 0.013] |
| Advance Payment or ACO Investment (ref: no participation) | 0.039 | [-0.160, | 0.237] |
| No. of assigned beneficiaries (1,000s) | 0.001** | [0.000, | 0.003] |
| No. of distinct participating organizations (100s) | -0.066*** | [-0.106, | -0.026] |
| Weighted HCC risk score | -0.414**** | [-0.618, | -0.210] |
| Beneficiary concentration in ACO service counties (HHI) | 0.191*** | [0.055, | 0.327] |
| Home health agency expense to total health expensea | 0.739** | [0.096, | 1.383] |
| **Social Determinants of Health** |  |  |  |
| Economic well-being |  |  |  |
| Unemployment ratea | -2.728*** | [-4.425, | -1.032] |
| Median household income ($10,000s) | 0.026 | [-0.011, | 0.063] |
| Transportation convenience |  |  |  |
| Commute by public transportationa | 1.836**** | [0.816, | 2.857] |
| Mean commute time (10 mins) | -0.139 | [-0.296, | 0.017] |
| Healthy food consumption |  |  |  |
| Low-income population with grocery store accessa | 5.567** | [0.782, | 10.353] |
| Per capita expenses on fast food ($100s) | -0.023 | [-0.855, | 0.808] |
| Health resource availability |  |  |  |
| Hospital-based registered nurse to bed ratio | 0.362*** | [0.129, | 0.596] |
| Observed/expected ratio of primary care checkups | 2.151**** | [1.504, | 2.797] |
| Average distance between healthcare organizations (10 miles) | 0.001 | [-0.021, | 0.023] |

**Source**: Authors’ analysis of data from the Medicare Shared Savings Program (MSSP) public use files, American Community Survey, Food Environment Atlas, Dartmouth Atlas of Healthcare, and Healthcare Information and Management Systems Society.

**Notes**: The model uses a full sample of 858 ACOs from 2014 to 2021 (3,625 ACO-year observations) that do not have missing covariates. R-squared equals 0.103 and F statistics equals 9.49. Joint significance tests are performed to examine each SDOH domain. The health resource availability factors are jointly significant (p<0.0001), while the transportation convenience and economic well-being factors are also jointly significant (p<0.001). The healthy food consumption factors are marginally significant (p=0.073). HCC is Hierarchical Condition Category. HHI is Herfindahl-Hirschman Index.

aCovariates are not in percent format for the ease of presenting regression coefficients.

**p < 0.05 ***p < 0.01 ****p < 0.001.

**Table F5. Regression results based on subsample of leadership type and ACO risk tracks, 2014 – 2021**

|  | (1) | (2) |
| --- | --- | --- |
| **Subsample** | ACOs that changed leadership type | ACOs that switched risk tracks |
|  |  |  |
| Hospital-managed (ref: physician-led) | 0.122**** | 0.079**** |
|  | [0.100, 0.143] | [0.046, 0.112] |
| Two-sided risk (ref: one-sided risk) | 0.030 | 0.027 |
|  | [-0.016, 0.077] | [-0.002, 0.055] |
|  |  |  |
| Observations | 801 | 1,047 |
| R-squared | 0.257 | 0.140 |
| F statistics | 6.62 | 4.23 |

**Source**: Authors’ analysis of data from the Medicare Shared Savings Program (MSSP) public use files, American Community Survey, Food Environment Atlas, Dartmouth Atlas of Healthcare, and Healthcare Information and Management Systems Society.

**Notes**: The model uses subsamples of 135 and 158 ACOs that changed leadership type or risk tracks, respectively. Other covariates of ACO characteristics and social determinants of health (SDOH) variables were included in the estimation model but not reported for the brevity of the table.

95% confidence intervals are presented in square brackets.

**p < 0.05 ***p < 0.01 ****p < 0.001.

**Table F6. Alternative regressions for social determinants of health (SDOH) factors and healthcare value, 2014 – 2021**

| **Variable** | **Regression Coefficient** | **95% Confidence Interval** | |
| --- | --- | --- | --- |
|  |  |  |  |
| **Social Determinants of Health** |  |  |  |
| Economic well-being |  |  |  |
| Unemployment ratea | -1.854**** | [-2.873, | -0.835] |
| Transportation convenience |  |  |  |
| Commute by public transportationa | 0.214 | [-0.355, | 0.784] |
| Healthy food consumption |  |  |  |
| Low-income population with grocery store accessa | 2.854** | [0.182, | 5.526] |
| Per capita expenses on fast food ($100s) | -0.013 | [-0.062, | 0.037] |
| Health resource availability |  |  |  |
| Hospital-based registered nurse to bed ratio | 0.128 | [-0.006, | 0.262] |
| Observed/expected ratio of primary care checkups | 0.635*** | [0.250, | 1.019] |
| Average distance between healthcare organizations (10 miles) | -0.010 | [-0.023, | 0.003] |

**Source**: Authors’ analysis of data from the Medicare Shared Savings Program (MSSP) public use files, American Community Survey, Food Environment Atlas, Dartmouth Atlas of Healthcare, and Healthcare Information and Management Systems Society.

**Notes**: The model uses a full sample of 858 ACOs from 2014 to 2021 (3,625 ACO-year observations) that do not have missing covariates. R-squared equals 0.107 and F statistics equals 10.58. Other covariates of ACO characteristics were included in the estimation model but not reported for the brevity of the table. Joint significance tests are performed to examine each SDOH domain. The healthy food consumption factors are marginally significant (p-value=0.094), while the health resource availability factors are jointly significant (p-value=0.001).

95% confidence intervals are presented in square brackets.

**p < 0.05 ***p < 0.01 ****p < 0.001.

**Appendix References**

1. Tone K. Variations on the theme of slacks-based measure of efficiency in DEA. *Eur J Oper Res*. 2010;200(3):901-907. doi:10.1016/j.ejor.2009.01.027

2. Cooper WW, Seiford LM, Zhu J. *Handbook on Data Envelopment Analysis*. Springer Science & Business Media; 2011.

3. Banker RD, Charnes A, Cooper WW. Some Models for Estimating Technical and Scale Inefficiencies in Data Envelopment Analysis. *Manag Sci*. 1984;30(9):1078-1092. doi:10.1287/mnsc.30.9.1078

4. Hollingsworth B. The measurement of efficiency and productivity of health care delivery. *Health Econ*. 2008;17(10):1107-1128. doi:10.1002/hec.1391

5. Li S, Shang J, Slaughter SA. Why Do Software Firms Fail? Capabilities, Competitive Actions, and Firm Survival in the Software Industry from 1995 to 2007. *Inf Syst Res*. 2010;21(3):631-654. doi:10.1287/isre.1100.0281

6. Demerjian P, Lev B, McVay S. Quantifying Managerial Ability: A New Measure and Validity Tests. *Manag Sci*. 2012;58(7):1229-1248. doi:10.1287/mnsc.1110.1487

7. Tiemann O, Schreyögg J. Changes in hospital efficiency after privatization. *Health Care Manag Sci*. 2012;15(4):310-326. doi:10.1007/s10729-012-9193-z

8. Tone K, Toloo M, Izadikhah M. A modified slacks-based measure of efficiency in data envelopment analysis. *Eur J Oper Res*. 2020;287(2):560-571. doi:10.1016/j.ejor.2020.04.019

9. Cantor VJM, Poh KL. Integrated analysis of healthcare efficiency: a systematic review. *J Med Syst*. 2018;42:1-23. doi:10.1007/s10916-017-0848-7

10. Kohl S, Schoenfelder J, Fügener A, Brunner JO. The use of Data Envelopment Analysis (DEA) in healthcare with a focus on hospitals. *Health Care Manag Sci*. 2019;22:245-286. doi:10.1007/s10729-018-9436-8

11. Bardhan IR, Bao C, Ayabakan S. Value Implications of Sourcing Electronic Health Records: The Role of Physician Practice Integration. *Inf Syst Res*. 2023;34(3):1169-1190. doi:10.1287/isre.2022.1183

12. Bleser WK, Saunders RS, Muhlestein DB, McClellan M. Why Do Accountable Care Organizations Leave The Medicare Shared Savings Program? *Health Aff (Millwood)*. 2019;38(5):794-803. doi:10.1377/hlthaff.2018.05097

13. Bao C, Bardhan IR. Performance of Accountable Care Organizations: Health Information Technology and Quality–Efficiency Trade-Offs. *Inf Syst Res*. 2022;33(2):697-717. doi:10.1287/isre.2021.1080

14. Lan Y, Chandrasekaran A, Goradia D, Walker D. Collaboration Structures in Integrated Healthcare Delivery Systems: An Exploratory Study of Accountable Care Organizations. *Manuf Serv Oper Manag*. 2022;24(3):1796-1820. doi:10.1287/msom.2021.1038

15. Colla CH, Lewis VA, Tierney E, Muhlestein DB. Hospitals Participating In ACOs Tend To Be Large And Urban, Allowing Access To Capital And Data. *Health Aff (Millwood)*. 2016;35(3):431-439. doi:10.1377/hlthaff.2015.0919

16. Colla CH, Lewis VA, Shortell SM, Fisher ES. First National Survey Of ACOs Finds That Physicians Are Playing Strong Leadership And Ownership Roles. *Health Aff (Millwood)*. 2014;33(6):964-971. doi:10.1377/hlthaff.2013.1463

17. NAACOS. Highlights of the 2020 Medicare ACO Program Results. Published 2022. Accessed October 27, 2023. https://www.naacos.com/highlights-of-the-2020-medicare-aco-program-results

18. Trombley MJ, McWilliams JM, Fout B, Morefield B. ACO Investment Model Produced Savings, But The Majority Of Participants Exited When Faced With Downside Risk. *Health Aff (Millwood)*. 2022;41(1):138-146. doi:10.1377/hlthaff.2020.01819

19. Lyu PF, Chernew ME, McWilliams JM. Benchmarking Changes And Selective Participation In The Medicare Shared Savings Program: *Health Aff (Millwood)*. 2023;42(5):622-631. doi:10.1377/hlthaff.2022.01061

20. Agarwal D, Werner RM. Effect of Hospital and Post‐Acute Care Provider Participation in Accountable Care Organizations on Patient Outcomes and Medicare Spending. *Health Serv Res*. 2018;53(6):5035-5056. doi:10.1111/1475-6773.13023

21. Lewis VA, Colla CH, Carluzzo KL, Kler SE, Fisher ES. Accountable Care Organizations in the United States: Market and Demographic Factors Associated with Formation. *Health Serv Res*. 2013;48(6pt1):1840-1858. doi:10.1111/1475-6773.12102

22. Islam MM. Social determinants of health and related inequalities: confusion and implications. *Front Public Health*. 2019;7:11. doi:10.3389/fpubh.2019.00011

23. McCarthy ML, Zheng Z, Wilder ME, Elmi A, Li Y, Zeger SL. The influence of social determinants of health on emergency departments visits in a Medicaid sample. *Ann Emerg Med*. 2021;77(5):511-522. doi:10.1016/j.annemergmed.2020.11.010

24. Davis CI, Montgomery AE, Dichter ME, Taylor LD, Blosnich JR. Social determinants and emergency department utilization: findings from the veterans health administration. *Am J Emerg Med*. 2020;38(9):1904-1909. doi:10.1016/j.ajem.2020.05.078

25. Guo T, Bardhan I, Khurshid A. Social Determinants of Health and ER Utilization: Role of Information Integration during COVID-19. *ACM Trans Manag Inf Syst*. 2023;14(4):1-25. doi:10.1145/3583077

26. Hardy R, Boch S, Keedy H, Chisolm D. Social determinants of health needs and pediatric health care use. *J Pediatr*. 2021;238:275-281. doi:10.1016/j.jpeds.2021.07.056

27. Ding X (David). Benchmark and performance progression: Examining the roles of market competition and focus. *J Oper Manag*.:1-30. doi:10.1002/joom.1288
